# PCSK9 inhibitor recaticimab for hypercholesterolemia on stable statin dose: a randomized, double-blind, placebo-controlled phase 1b/2 study

**DOI:** 10.1186/s12916-021-02208-w

**Published:** 2022-01-18

**Authors:** Mingtong Xu, Xiaoxue Zhu, Junyan Wu, Yuling Zhang, Dong Zhao, Xuhong Wang, Yanhua Ding, Yu Cao, Chengqian Li, Wei Hu, Jianlong Sheng, Zhu Luo, Zeqi Zheng, Jinfang Hu, Jianying Liu, Xiaoyang Zhou, Aizong Shen, Xiaomei Ding, Yongdong Zhang, Yonggang Zhao, Yijing Li, Sheng Zhong, Shimin An, Jianjun Zou, Li Yan

**Affiliations:** 1 Department of Endocrinology, Sun Yat-Sen Memorial Hospital, Sun Yat-Sen University, No. 107 Yanjiang Road West, Guangzhou, 510120 China; 2 Phase I Clinical Trials Unit, The First Hospital of Jilin University, Changchun, China; 3 Department of Pharmacy, Sun Yat-Sen Memorial Hospital, Sun Yat-Sen University, Guangzhou, China; 4 Department of Cardiology, Sun Yat-Sen Memorial Hospital, Sun Yat-Sen University, Guangzhou, China; 5 Endocrinology Center, Capital Medical University, Beijing Luhe Hospital, Beijing, China; 6 Phase I Clinical Research Center, The Affiliated Hospital of Qingdao University, Qingdao, China; 7 Department of Endocrinology, The Affiliated Hospital of Qingdao University, Qingdao, China; 8 Department of Pharmacology, The Second Hospital of Anhui Medical University, Hefei, China; 9 Department of Cardiology, The Second Affiliated Hospital of Anhui Medical University, Hefei, China; 10 Department of Clinical Laboratory Medicine, West China Hospital, Sichuan University, Chengdu, China; 11 Department of Cardiology, The First Affiliated Hospital of Nanchang University, Nanchang, China; 12 Department of Pharmacy, The First Affiliated Hospital of Nanchang University, Nanchang, China; 13 Department of Endocrinology and Metabolism, The First Affiliated Hospital of Nanchang University, Nanchang, China; 14 Department of Cardiology, Renmin Hospital of Wuhan University, Wuhan, China; 15 Department of Pharmacy, Anhui Provincial Hospital Affiliated to Anhui Medical University, Hefei, China; 16 Department of Cardiology, Anhui Provincial Hospital Affiliated to Anhui Medical University, Hefei, China; 17 Department of Pharmacy, Chenzhou First People’s Hospital, Chenzhou, China; 18 Department of Emergency Medicine, Chenzhou First People’s Hospital, Chenzhou, China; 19 Clinical Research & Development, Jiangsu Hengrui Pharmaceuticals Co., Ltd., Shanghai, China

**Keywords:** PCSK9, Hypercholesterolemia, Recaticimab, Infrequent administration

## Abstract

**Background:**

Recaticimab (SHR-1209, a humanized monoclonal antibody against PCSK9) showed robust LDL-C reduction in healthy volunteers. This study aimed to further assess the efficacy and safety of recaticimab in patients with hypercholesterolemia.

**Methods:**

In this randomized, double-blind, placebo-controlled phase 1b/2 trial, patients receiving stable dose of atorvastatin with an LDL-C level of 2.6 mmol/L or higher were randomized in a ratio of 5:1 to subcutaneous injections of recaticimab or placebo at different doses and schedules. Patients were recruited in the order of 75 mg every 4 weeks (75Q4W), 150Q8W, 300Q12W, 150Q4W, 300Q8W, and 450Q12W. The primary endpoint was percentage change in LDL-C from the baseline to end of treatment (i.e., at week 16 for Q4W and Q8W schedule and at week 24 for Q12W schedule).

**Results:**

A total of 91 patients were enrolled and received recaticimab and 19 received placebo. The dose of background atorvastatin in all 110 patients was 10 or 20 mg/day. The main baseline LDL-C ranged from 3.360 to 3.759 mmol/L. The least-squares mean percentage reductions in LDL-C from baseline to end of treatment relative to placebo for recaticimab groups at different doses and schedules ranged from −48.37 to −59.51%. No serious treatment-emergent adverse events (TEAEs) occurred. The most common TEAEs included upper respiratory tract infection, increased alanine aminotransferase, increased blood glucose, and increased gamma-glutamyltransferase.

**Conclusion:**

Recaticimab as add-on to moderate-intensity statin therapy significantly and substantially reduced the LDL-C level with an infrequent administration schedule (even given once every 12 weeks), compared with placebo.

**Trial registration:**

ClinicalTrials.gov, number NCT03944109

**Supplementary Information:**

The online version contains supplementary material available at 10.1186/s12916-021-02208-w.

## Background

Hypercholesterolemia, a common disorder characterized by an elevated plasma concentration of low-density lipoprotein-cholesterol (LDL-C), is one of the predominant causes for atherosclerosis and, consequently, cardiovascular disease. Reducing the level of LDL-C remains the cornerstone of hypercholesterolemia management and atherosclerotic cardiovascular disease prevention [1, 2]. A large-scale meta-analysis found that each 1.0 mmol/L reduction in LDL-C level leads to a 22% decrease in the annual rate of major vascular events [3].

Statins that work as cholesterol-synthesis inhibitors are widely prescribed lipid-lowering drugs, with approximately 20 to 65% reduction in the LDL-C level [4]. However, many patients are unable to achieve sufficient LDL-C lowering due to insufficient response, treatment resistance, and/or drug intolerance mainly in the terms of muscle-related symptoms and liver enzyme abnormalities [4–8]. Myopathy and consecutive alanine transaminase >3× upper limit of normal are even more common among population in China compared with Europe [9]. In addition, doubling of the statin dose only leads to an LDL-C reduction of 6% and increases the risk of toxicity [10]. As such, adjunctive therapies adding to a statin are required. The addition of gut-acting drugs (bile acid sequestrants or ezetimibe) to a statin regimen only produces an additional 13 to 30% reduction in LDL-C, and bile acid sequestrants are associated with headaches and gastrointestinal complaints, which limit their practical use [2].

Proprotein convertase subtilisin/kexin type 9 (PCSK9) is a ubiquitous serine protease with pleiotropic tissue-specific functions; among them, the most characterized one is its modulation effect on lipid metabolism [11]. PCSK9 predominantly expresses in the liver and secretes into the plasma. By inhibiting the recycling of hepatic LDL receptors, serum PCSK9 leads to increase in LDL-C concentration and consequently high risk of cardiovascular events [12–14]. The two worldwide approved monoclonal antibodies against PCSK9 (evolocumab and alirocumab) for hypercholesterolemia could further increase the magnitude of LDL-C lowering by up to 60%, when administrated as add-on to a statin [2, 15–17], accompanied by a reduced risk of cardiovascular events [18–20] and improved the quality of life [21]. But subcutaneous injection every 2 or 4 weeks is a big challenge for patient adherence.

Recaticimab (SHR-1209) is a humanized immunoglobulin G1 monoclonal antibody that binds PCSK9 with high affinity. A phase 1a randomized, double-blind, placebo-controlled study in healthy volunteers indicated that a single dose of 51 to 450 mg recaticimab is safe and well-tolerated and produces a 50 to 65% reduction in serum LDL-C level (ClinicalTrials.gov identifier NCT03634436; data on file, Jiangsu Hengrui Pharmaceuticals). Here, in this phase 1b/2 study, we reported the efficacy, safety, and pharmacokinetics of recaticimab at different doses and frequencies in hypercholesterolemia patients on background statin therapy, as well as the immunogenicity of recaticimab.

## Methods

### Participants

This was a randomized, double-blind, placebo-controlled phase 1b/2 trial of recaticimab or placebo in patients with hypercholesterolemia on stable atorvastatin, which was done at 11 study sites in China. Patients were eligible if they were adults 18 to 65 years of age with an LDL-C level of 2.6 mmol/L or higher before randomization (either for those receiving statin therapy and/or other lipid-lowering treatments and had an LDL-C level of 2.6 mmol/L or higher at screening, or for those who had not previously received any lipid-lowering treatment and had an LDL-C level of 3.4 mmol/L or higher at screening), a fasting triglyceride (TG) level of 4.5 mmol/L or lower, and a body mass index of 18 to 35 kg/m^2^. Patients with homozygous familial hypercholesterolemia were excluded from the study. The full inclusion and exclusion criteria are available in Additional file 1.

The study was approved by the ethics committee at each study site and conducted according to the Declaration of Helsinki, Guidelines for Good Clinical Practice, and local laws and regulations. All patients provided written informed consent. The study was registered on ClinicalTrials.gov, number NCT03944109.

### Study design

This study included a screening and statin run-in period, a treatment period, and a 12-week follow-up period (Additional file: Figure S1). All patients were required to receive a stable regimen of atorvastatin (10 to 40 mg/day) for at least 28 days before randomization and continue the regimen throughout the study.

Eligible patients who had an LDL-C level of 2.6 mmol/L or higher at screening or after the run-in period were assigned to different doses and schedules of treatment in the order of 75 mg every 4 weeks (75Q4W), 150 mg every 8 weeks (150Q8W), 300 mg every 12 weeks (300Q12W), 150 mg every 4 weeks (150Q4W), 300 mg every 8 weeks (300Q8W), and 450 mg every 12 weeks (450Q12W). At each allocated dose and schedule, patients were randomized, in a 5:1 ratio, to receive recaticimab or matching placebo by using a centralized interactive web-response system with no stratification factors. Either recaticimab or placebo was administrated subcutaneously in the abdominal area for 16 weeks for patients with Q4W and Q8W schedules or 24 weeks for patients with Q12W schedule, followed by a 12-week follow-up. Patients, investigators, study site staff, and the sponsor were masked to treatment assignment until study completion.

### Outcomes and assessments

The primary endpoint was percentage change in LDL-C from baseline to end of treatment: at week 16 for patients receiving treatment Q4W and Q8W and at week 24 for patients receiving treatment Q12W. Secondary efficacy endpoints included absolute change in LDL-C from baseline to end of treatment, percentage changes from baseline to end of treatment in other lipids (including total cholesterol [TC], high-density lipoprotein-cholesterol [HDL-C], non-HDL-C, and TG), apolipoproteins (including ApoB, ApoA1, and Lp[a]), and free PCSK9. Changes in these parameters was also measured at each scheduled visit. All lipid and apolipoprotein variables were centrally measured by the KingMed Diagnostics Group (Guangzhou, China), and free PCSK9 was centrally measured by the Frontage Biotechnology (Shanghai, China).

Safety was assessed by treatment-emergent adverse events (TEAEs), vital signs, physical examination, 12-lead electrocardiograph, laboratory tests, and injection-site reaction.

Pharmacokinetics and detection of binding antidrug antibodies (ADAs) were evaluated after the first and last administration. Testing for neutralizing antibodies was conducted for all positive tests for binding ADAs.

### Statistical analysis and sample size calculation

The sample size was calculated based on the assumption that the percentage reduction in LDL-C from baseline to end of treatment in each dose and schedule of recaticimab group would be 30% higher than that in the placebo group (including patients treated with placebo at all doses and schedules). We calculated that approximately 108 patients (including 10 patients in each of recaticimab 75Q4W, 150Q8W, and 300Q12W groups, 20 in each of recaticimab 150Q4W, 300Q8W, and 450Q12W groups, and 18 in placebo group) would provide at least 90% power to detect the treatment difference between each recaticimab group and placebo group, assuming a standard deviation (SD) of 20%, and using a two-sample *t* test at the 0.05 significance level.

Efficacy and pharmacokinetics analyses were done in all randomized patients who received at least one dose of study treatment and had baseline and at least one post-baseline assessment. Safety and immunogenicity were analyzed in all randomized patients who received at least one dose of study treatment.

Treatment differences on percentage or absolute change in LDL-C from baseline to end of treatment were performed using analysis of ANCOVA models, and the missing data were imputed using the last observation carried forward method. Least-squares (LS) mean with 95% confidence intervals (CIs) were calculated for each group, and LS mean difference and corresponding 95% CIs between recaticimab treatment group and placebo were also provided with *p* values for pairwise comparisons. Descriptive statistical analyses were used to summarize other secondary efficacy endpoints, and no imputation was used for missing values. Adherence to treatment was calculated as the actual total dose divided by the total prescribed dose. Efficacy analyses were done with SAS version 9.2.

The serum concentration-time profiles of recaticimab at different doses and schedules were graphically presented. Individual plasma pharmacokinetics parameters were estimated using non-compartmental methods by Phoenix WinNonlin (Version 8.0), and descriptive statistical analyses were used to report pharmacokinetics and immunogenicity parameters.

## Results

### Study population

**Fig. 1:**
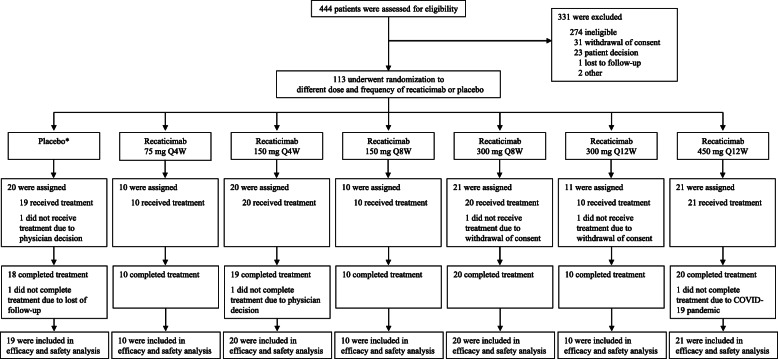
Study flow. *2, 2, 2, 4, 4, and 4 patients were assigned to receive placebo at a dose and frequency of 75 mg Q4W, 150 mg Q4W, 150 mg Q8W, 300 mg Q8W, 300 mg Q12W, and 450 mg Q12W, respectively

Between July 31, 2019, and June 16, 2020, 444 patients were screened; of these patients, 113 were eligible and underwent randomization to receive different doses and schedules of recaticimab or matching placebo, on background atorvastatin therapy (Fig. 1). Three patients did not receive the allocated intervention after randomization and thus were excluded from efficacy and safety analyses, including one patient in the placebo group, one in the recaticimab 300Q8W group, and one in the recaticimab 300Q12W group. Of the 110 patients who received study treatment, 107 (97.3%) completed preplanned treatment period, whereas only three patients stopped treatment early due to loss to follow-up, physician decision, and COVID-19 pandemic, respectively.

The dose of background atorvastatin in all 110 patients was 10 or 20 mg/d. Patients in the recaticimab groups showed well compliance to treatment, with 97.8% (89/91) of patients taking 80 to 120% of the prescribed atorvastatin and 95.0 to 100% of patients taking at least 80% of the prescribed recaticimab at different dosing schedules (100% [10/10], 95.0% [19/20], 100% [10/10], 100% [20/20], 100% [10/10], and 95.2% [20/21] at 75Q4W, 150Q4W, 150Q8W, 300Q8W, 300Q12W, and 450Q12W, respectively).

Baseline characteristics were generally similar between the recaticimab groups and the placebo group, except a little higher proportion of females in the placebo group (Additional file: Table S1). The mean age of patients was 50.3 years (range, 22 to 65; SD, 9.48).

### Efficacy

Compared with placebo, all doses and schedules of recaticimab groups achieved a significant decrease in the primary efficacy endpoint of percentage change in LDL-C from baseline to end of treatment (Table 1). The LS mean percentage reductions in LDL-C were −48.63% (95% CI −59.80, −37.46), −55.06% (−62.96, −47.17), −52.02% (−63.12, −40.86), −48.38% (−56.32, −40.44), −43.93% (−55.10, −32.77), and −52.77% (−60.49, −45.05) in the recaticimab 75Q4W, 150Q4W, 150Q8W, 300Q8W, 300Q12W, and 450Q12W groups, respectively, as compared with an increase of 4.44% (−3.69, 12.57) in the placebo group (all *p* values <0.0001). The LS mean percentage reductions in LDL-C relative to placebo for recaticimab groups ranged from −48.37 to −59.51%. The LS mean absolute reductions in LDL-C relative to placebo ranged from −1.59 to −1.92 mmol/L in recaticimab groups.

**Table 1:** Change in LDL-C from baseline to end of treatment

	Placebo (***N***=19)	Recaticimab					
		75 mg Q4W (***N***=10)	150 mg Q4W (***N***=20)	150 mg Q8W (***N***=10)	300 mg Q8W (***N***=20)	300 mg Q12W (***N***=10)	450 mg Q12W (***N***=21)
**Baseline (mmol/L), mean (SD)**	3.360 (0.928)	3.656 (0.951)	3.563 (0.900)	3.464 (0.753)	3.759 (0.874)	3.608 (0.674)	3.420 (0.835)
**Percentage change (%)**							
LS mean (95% CI)	4.44 (−3.69, 12.57)	−48.63 (−59.80, −37.46)	−55.06 (−62.96, −47.17)	−52.02 (−63.12, −40.86)	−48.38 (−56.32, −40.44)	−43.93 (−55.10, −32.77)	−52.77 (−60.49, −45.05)
Change relative to placebo, LS mean (95% CI); *p*	..	−53.07 (−66.91, −39.23); *p*<0.0001	−59.51 (−70.84, −48.17); *p*<0.0001	−56.46 (−70.26, −42.67); *p*<0.0001	−52.82 (−64.24, −41.40); *p*<0.0001	−48.37 (−62.20, −34.55); *p*<0.0001	−57.21 (−68.39, −46.04); *p*<0.0001
**Absolute change (mmol/L)**							
LS mean (95% CI)	0.02 (−0.27, 0.31)	−1.69 (−2.09, −1.29)	−1.9 (−2.18, −1.62)	−1.84 (−2.24, −1.44)	−1.73 (−2.01, −1.44)	−1.57 (−1.98, −1.17)	−1.85 (−2.13, −1.58)
Change relative to placebo, LS mean (95% CI); *p*	..	−1.71 (−2.21, −1.22); *p*<0.0001	−1.92 (−2.33, −1.51); *p*<0.0001	−1.86 (−2.36, −1.37); *p*<0.0001	−1.75 (−2.16, −1.34); *p*<0.0001	−1.59 (−2.09, −1.10); *p*<0.0001	−1.87 (−2.28, −1.47); *p*<0.0001

*LDL-C* low-density lipoprotein-cholesterol, *SD* standard deviation, *LS* least-squares, *CI* confidence interval

**Fig. 2:**
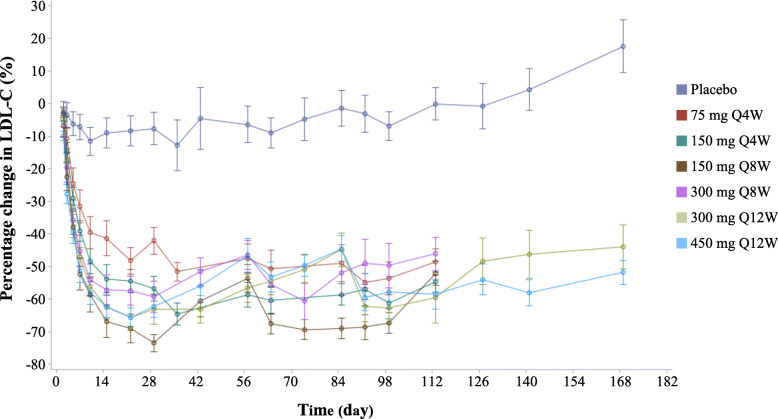
Percentage change in LDL-C during treatment. Mean percentage changes (standard error) in LDL-C from baseline to end of treatment (i.e., at week 16 for patients receiving treatment Q4W and Q8W and at week 24 for patients receiving treatment Q12W) are shown. LDL-C low-density lipoprotein cholesterol

Figure 2 shows the mean percentage change in LDL-C concentration during whole treatment period. Rapid reductions in LDL-C were observed after the initial administration in all recaticimab groups. During the first cycle, the maximum reduction in LDL-C level occurred at week 3 for 75Q4W (−48.18%) and at week 4 for 150Q4W (−56.74%); thereafter, the percentage change maintained −42.11 to −54.92% in the recaticimab 75Q4W group and −54.58 to −64.53% in the recaticimab 150Q4W group. The maximum reduction in LDL-C occurred at week 4 for 150Q8W (−73.52%) and 300Q8W (−59.27%) and week 3 for 300Q12W (−65.72%) and 450Q12W (−65.52%) during the first cycle; thereafter, there was a slight rebound before the next administration; however, substantial reduction persisted, ranging from −52.05 to −69.32% for 150Q8W, −46.13 to −60.48% for 300Q8W, −43.91 to −63.11% for 300Q12W, and −44.49 to −59.56% for 450Q12W.

The percentage decreases in other lipid and apolipoprotein measures are summarized in Table 2. Rapid and sustained reductions in TC, non-HDL-C, ApoB, and Lp(a) were noted in all recaticimab groups (Additional file: Figure S2-S5). The percentage change in TC from baseline to end of treatment ranged from −31.263 to −37.760% in recaticimab groups, as compared with 3.534 to −3.065% with placebo. The percentage change in non-HDL-C from baseline to end of treatment ranged from −39.920 to −52.349% in recaticimab groups, as compared with 7.412 to −5.957% with placebo. The percentage change in ApoB from baseline to end of treatment ranged from −35.592 to −47.687% in recaticimab groups, as compared with 12.565% to −6.623% with placebo. The percentage change in Lp(a) from baseline to end of treatment ranged from −21.612 to −47.521% in recaticimab groups, as compared with −7.505 to −15.471% with placebo. No meaningful changes were found in HDL-C, TG, and ApoA1 levels (Additional file: Figure S6-S8).

**Table 2:** Decrease in lipid and apolipoprotein measures from baseline to end of treatment

	Placebo Q4W (***N***=6)	75 mg Q4W (***N***=10)	150 mg Q4W (***N***=20)	Placebo Q8W (***N***=6)	150 mg Q8W (***N***=10)	300 mg Q8W (***N***=20)	Placebo Q12W (***N***=7)	300 mg Q12W (***N***=10)	450 mg Q12W (***N***=21)
**TC**									
Baseline (mmol/L)	4.570 (0.165)	5.169 (0.329)	5.352 (0.167)	5.223 (0.358)	5.304 (0.271)	5.455 (0.174)	5.830 (0.473)	5.186 (0.232)	5.081 (0.191)
Percentage change from baseline to end of treatment (%)	1.578 (5.877)	−32.940 (3.048)	−34.162 (3.234)	−3.065 (4.836)	−37.760 (3.432)	−31.576 (3.516)	3.534 (5.057)	−31.263 (5.005)	−35.339 (3.186)
**Non-HDL-C**									
Baseline (mmol/L)	3.220 (0.241)	4.116 (0.304)	4.071 (0.170)	4.020 (0.399)	4.032 (0.225)	4.281 (0.183)	4.179 (0.360)	4.029 (0.223)	3.780 (0.182)
Percentage change from baseline to end of treatment (%)	2.746 (8.305)	−46.991 (3.605)	−49.979 (4.396)	−5.957 (6.166)	−52.349 (3.301)	−39.920 (4.441)	7.412 (6.497)	−43.542 (6.503)	−47.383 (3.900)
**ApoB**									
Baseline (g/L)	0.908 (0.065)	1.151 (0.070)	1.112 (0.050)	1.115 (0.095)	1.094 (0.059)	1.194 (0.053)	1.191 (0.085)	1.106 (0.058)	1.072 (0.046)
Percentage change from baseline to end of treatment (%)	7.087 (7.955)	−43.392 (3.719)	−47.026 (4.180)	−6.623 (6.839)	−47.687 (3.313)	−39.937 (4.397)	12.565 (6.040)	−35.592 (6.504)	−41.838 (3.490)
**Lp(a)**									
Baseline (g/L)	8.200 (3.550)	9.859 (2.603)	11.285 (2.671)	2.776 (0.724)	16.416 (6.548)	13.424 (4.407)	36.285 (16.171)	16.013 (6.034)	17.401 (4.244)
Percentage change from baseline to end of treatment (%)	−8.543 (4.712)	−34.074 (5.678)	−41.434 (7.972)	−15.471 (16.595)	−21.612 (9.532)	−47.521 (5.777)	−7.505 (7.441)	−34.300 (8.296)	−27.557 (6.045)

Data are shown in mean (standard error)

*TC* total cholesterol, *Apo B* apolipoprotein B, *HDL-C* high-density lipoprotein cholesterol, *Lp(a)* lipoprotein a

Sharp and dose-dependent percentage reduction in circulating free PCSK9 with recaticimab treatment were detected immediately after administration (Additional file: Figure S9). The maximum suppression of free PCSK9 after first administration was −31.60% with recaticimab 75Q4W and −44.35% with recaticimab 150Q4W as compared with −9.57% with placebo Q4W, −47.49% with recaticimab 150Q8W and −70.20% with recaticimab 300Q8W as compared with −10.17% with placebo Q8W, and −67.47% with recaticimab 300Q12W and −74.50% with recaticimab 450Q12W as compared with −16.57% with placebo Q12W. Rebound was observed; however, the free PCSK9 level was always below baseline level before next administration in the recaticimab 75Q4W, 150Q4W, and 300Q8W groups, while the free PCSK9 level increased to higher than baseline level near the next administration in the recaticimab 150Q8W, 300Q12W, and 450Q12W groups.

### Safety assessments

Overall, the incidences of TEAEs were similar in patients who received recaticimab (74.7% [68/91]) or placebo (73.7% [14/19]). The most common TEAEs included upper respiratory tract infection (19.8% with recaticimab versus 15.8% with placebo), increased alanine aminotransferase (9.9% versus 10.5%), increased blood glucose (8.8% versus 0), and increased gamma-glutamyltransferase (6.6% versus 0; Table 3).

**Table 3:** Safety data

	Placebo (***N***=19)	Recaticimab						
		75 mg Q4W (***N***=10)	150 mg Q4W (***N***=20)	150 mg Q8W (***N***=10)	300 mg Q8W (***N***=20)	300 mg Q12W (***N***=10)	450 mg Q12W (***N***=21)	All patients with recaticimab (***N***=91)
**Any TEAEs**	14 (73.7%)	5 (50.0%)	18 (90.0%)	9 (90.0%)	14 (70.0%)	5 (50.0%)	17 (81.0%)	68 (74.7%)
Mild	14 (73.7%)	5 (50.0%)	15 (75.0%)	8 (80.0%)	14 (70.0%)	5 (50.0%)	15 (71.4%)	62 (68.1%)
Moderate	0	0	2 (10.0%)	1 (10.0%)	0	0	2 (9.5%)	5 (5.5%)
Severe	0	0	1 (5.0%)	0	0	0	0	1 (1.1%)
**TEAEs occurring in at least 5% of patients who received recaticimab or placebo**								
Upper respiratory tract infection	3 (15.8%)	1 (10.0%)	7 (35.0%)	1 (10.0%)	3 (15.0%)	1 (10.0%)	5 (23.8%)	18 (19.8%)
Alanine aminotransferase increased	2 (10.5%)	0	2 (10.0%)	2 (20.0%)	2 (10.0%)	2 (20.0%)	1 (4.8%)	9 (9.9%)
Blood glucose increased	0	0	1 (5.0%)	1 (10.0%)	2 (10.0%)	1 (10.0%)	3 (14.3%)	8 (8.8%)
Gamma-glutamyltransferase increased	0	0	3 (15.0%)	0	3 (15.0%)	0	0	6 (6.6%)
White blood cell count increased	1 (5.3%)	0	1 (5.0%)	0	2 (10.0%)	0	2 (9.5%)	5 (5.5%)
White blood cells urine positive	1 (5.3%)	0	0	0	3 (15.0%)	0	2 (9.5%)	5 (5.5%)
Injection-site reaction	0	1 (10.0%)	0	0	3 (15.0%)	0	1 (4.8%)	5 (5.5%)
Protein urine present	0	0	2 (10.0%)	0	0	0	3 (14.3%)	5 (5.5%)
Aspartate aminotransferase increased	1 (5.3%)	0	2 (10.0%)	1 (10.0%)	0	2 (20.0%)	0	5 (5.5%)
Blood bilirubin increased	1 (5.3%)	0	0	2 (20.0%)	1 (5.0%)	0	1 (4.8%)	4 (4.4%)
Neutrophil count increased	1 (5.3%)	0	1 (5.0%)	0	1 (5.0%)	0	2 (9.5%)	4 (4.4%)
Hyperuricaemia	1 (5.3%)	1 (10.0%)	1 (5.0%)	0	1 (5.0%)	0	1 (4.8%)	4 (4.4%)
Hypertension	1 (5.3%)	0	0	2 (20.0%)	0	0	2 (9.5%)	4 (4.4%)
Urinary tract infection	1 (5.3%)	0	0	0	0	0	3 (14.3%)	3 (3.3%)
Pharyngitis	2 (10.5%)	0	1 (5.0%)	0	0	0	1 (4.8%)	2 (2.2%)
Urine leukocyte esterase positive	1 (5.3%)	0	1 (5.0%)	0	1 (5.0%)	0	0	2 (2.2%)
Red blood cells urine positive	1 (5.3%)	0	1 (5.0%)	0	0	0	1 (4.8%)	2 (2.2%)
Blood creatinine increased	1 (5.3%)	0	0	2 (20.0%)	0	0	0	2 (2.2%)
Dizziness	1 (5.3%)	1 (10.0%)	0	0	0	0	1 (4.8%)	2 (2.2%)
Hematuria	1 (5.3%)	0	0	0	0	0	2 (9.5%)	2 (2.2%)
Urinary sediment present	1 (5.3%)	0	1 (5.0%)	0	0	0	0	1 (1.1%)
Blood creatine phosphokinase increased	1 (5.3%)	0	1 (5.0%)	0	0	0	0	1 (1.1%)
Blood pressure increased	1 (5.3%)	0	0	0	0	1 (10.0%)	0	1 (1.1%)
Type 2 diabetes mellitus	1 (5.3%)	0	0	0	0	0	1 (4.8%)	1 (1.1%)
Muscle strain	1 (5.3%)	1 (10.0%)	0	0	0	0	0	1 (1.1%)
Anemia	1 (5.3%)	0	1 (5.0%)	0	0	0	0	1 (1.1%)
Urinary casts present	1 (5.3%)	0	0	0	0	0	0	0
Prothrombin time ratio increased	1 (5.3%)	0	0	0	0	0	0	0
Gastroenteritis	1 (5.3%)	0	0	0	0	0	0	0
Vaginal infection	1 (5.3%)	0	0	0	0	0	0	0
Pyrexia	1 (5.3%)	0	0	0	0	0	0	0
Arthralgia	1 (5.3%)	0	0	0	0	0	0	0
Supraventricular extrasystoles	1 (5.3%)	0	0	0	0	0	0	0
Leukocyturia	1 (5.3%)	0	0	0	0	0	0	0
Insomnia	1 (5.3%)	0	0	0	0	0	0	0

Data are shown in *n* (%)

*TEAE* treatment-emergent adverse events

There were no serious TEAEs or TEAEs leading to permanent discontinuation of treatment or death

Despite a high incidence of TEAEs, majority of TEAEs were mild (68.1% of patients treated with recaticimab versus 73.7% of patients with placebo). Five (5.5%) patients in the recaticimab groups had moderate TEAEs. Among them, 3 (3.3%) patients had moderate TEAEs that were deemed related to treatment, including increased alanine aminotransferase, increased gamma-glutamyltransferase, increased aspartate aminotransferase, herpes simplex, and ventricular extrasystoles (each occurred in one [1.1%] patient; Additional file: Table S2). Only one (1.1%) patient in the recaticimab groups had a severe TEAE (increased gamma-glutamyltransferase) that was deemed related to the study treatment by the investigator. All moderate and severe treatment-related TEAEs were improved or resolved by symptomatic treatments or interruption of study treatment, except the outcome of a ventricular extrasystole was unknown due to end of study.

During study, no patients experienced serious TEAEs, and no TEAEs leading to treatment discontinuation or death occurred. Injection-site reactions were reported in five (5.5%) patients with recaticimab.

### Pharmacokinetics

Plasma concentration-time curve is shown in Additional file (Figure S10). After first administration of recaticimab, the median time to reach maximum serum concentration (T_max_) ranged from 6.0 to 9.0 days, and no dose-dependent manner was observed (Table 4). With the same administration schedule, the maximum serum concentration (C_max_) was remarkably increased with increasing dose (geometric mean, 12.1 μg/mL at 150Q4W versus 5.7 μg/mL at 75Q4W, 31.4 μg/mL at 300Q8W versus 9.8 μg/mL at 150Q8W, and 43.2 μg/mL at 450Q12W and 28.4 μg/mL at 300Q12W). At the same dose, the C_max_ was only slightly decreased with prolonged administration schedule. Area under the plasma concentration-time profile from time zero to the treatment (AUC_0-τ_) and to the last concentration measurement (AUC_last_) showed a similar trend (Table 4).

**Table 4:** Pharmacokinetic parameters for recaticimab

	75 mg Q4W (***N***=10)	150 mg Q4W (***N***=17)	150 mg Q8W (***N***=10)	300 mg Q8W (***N***=15)	300 mg Q12W (***N***=10)	450 mg Q12W (***N***=20)
**After first dose**						
T_max_ (day)	9.0 (3.9, 14.0)	9.0 (6.0, 20.9)	7.0 (4.0, 14.0)	8.9 (2.0, 22.0)	9.0 (6.0, 21.0)	6.0 (3.9, 22.0)
C_max_ (μg/mL)	5.7 (43.7%)	12.1 (35.9%)	9.8 (41.0%)	31.4 (23.6%)	28.4 (44.0%)	43.2 (39.9%)
AUC_0-τ_ (day·μg/mL)	126 (45%)	260 (41%)	299 (37%)	1010 (26%)	1180 (40%)	1640 (46%)
AUC_last_ (day·μg/mL)	123 (42%)	258 (37%)	299 (37%)	1010 (26%)	1180 (40%)	1640 (46%)
**After last dose**						
T_max_ (day)	7.0 (5.9, 15.0)	7.0 (3.9, 28.0)	7.0 (3.0, 17.0)	7.0 (3.9, 32.2)	7.5 (7.0, 14.0)	7.0 (4.0, 28.9)
C_max_ (μg/mL)	8.7 (42.5%)	24.4 (33.9%)	13.0 (62.7%)	31.7 (41.9%)	32.0 (32.4%)	41.1 (27.0%)
t_1/2_ (day)	22.0 (27.3%)	22.1 (28.0%)	18.6 (11.8%)	21.7 (25.7%)	19.9 (24.8%)	27.4 (33.2%)
AUC_0-τ_ (day·μg/mL)	197 (44%)	553 (37%)	411 (54%)	1130 (47%)	1150 (31%)	1770 (36%)
AUC_last_ (day·μg/mL)	327 (59%)	988 (41%)	460 (55%)	1310 (48%)	1150 (31%)	1760 (36%)
AUC_0-∞_ (day·μg/mL)	367 (56%)	1090 (47%)	487 (53%)	1430 (51%)	1300 (38%)	2090 (43%)
CL/F (L/day)	0.4 (44.4%)	0.3 (37.2%)	0.4 (54%)	0.3 (47.1%)	0.3 (30.6%)	0.3 (36.1%)
Vz/F (L)	12.0 (33.8%)	8.4 (30.2%)	9.8 (56.4%)	8.3 (50.2%)	7.2 (18.6%)	10.1 (28.6%)
C_trough_ (μg/mL)	4.8 (53.8%)	13.4 (46.1%)	1.9 (45.8%)	7.5 (61.2%)	3.0 (97.8%)	4.0 (111.1%)

Data are median (range) for T_max_ and geometric mean (CV%) for other parameters

*CV* coefficient of variation, *T*_*max*_ time to reach maximum serum concentration, *C*_*max*_ maximum serum concentration, *AUC*_*0-τ*_ area under the plasma concentration-time profile from time zero to the treatment, *AUC*_*last*_ AUC from time zero to the last concentration measurement, *AUC*_*0-∞*_ AUC from time zero to infinity, *t1/2* half-life, *CL/F* apparent total clearance, *Vz/F* apparent volume of distribution, and *C*_*trough*_ trough concentration

After the last administration of recaticimab, the median T_max_ was 7.0 to 7.5 days, and exposure of recaticimab increased in a dose-dependent manner (Table 4). The t_1/2_ was similar with recaticimab treatment at different doses and schedules, except the 450Q12W (geometric mean, 27.4 days with 450Q12W; 18.6 to 22.1 days with other doses and schedules). No obvious differences were observed in apparent total clearance (0.3 to 0.4 L/day) or apparent volume of distribution (7.2 to 12.0 L).

### Immunogenicity

In our study, 26.4% (24/91) of the patients treated with recaticimab had detectable ADAs, but further analysis showed that the serum exposure of recaticimab were similar between patients who developed and those who did not develop ADAs (Additional file: Figure S11). Neutralizing antibodies only developed in 1.1% (1/91) of patients.

## Discussion

In the current phase 1b/2 study, administration of recaticimab at all tested doses and schedules provided a rapid and sustained LDL-C reduction in patients with hypercholesterolemia on stable statin dose.

It has been reported that treatment with atorvastatin increases the level of plasma PCSK9, which partly explained why increasing statin doses only led to diminished reduction in LDL-C level [22, 23]. By binding PCSK9, recaticimab could attenuate the interaction of PCSK9 with the LDL receptors, supporting that addition of recaticimab to statin therapy may result in even further LDL-C decreases. As expected, the LS mean percentage reductions in LDL-C from baseline to end of treatment were −48.63% and −55.06% in the recaticimab 75Q4W and 150Q4W groups, as compared with an increase of 4.44% in the placebo group (*p* values <0.0001). The reduction levels with recaticimab at Q4W dosing schedule were similar to those of alirocumab or evolocumab (−41.8 to −66.1% with alirocumab 75–150 mg Q2W or 300 mg every 4 weeks and evolocumab 140 mg every 2 weeks or 420 mg Q4W) [2, 15–17, 24, 25].

In clinical trials, patient adherence seemed to be not influenced by dosing schedules; however, in clinical practice, durability of reductions in LDL-C with lipid-lowering medications that require frequent dosing are reliant on patient adherence, and poor adherence may associate with worse outcomes. For a long time, there are only two worldwide approved PCSK9 inhibitors (i.e., alirocumab and evolocumab) that are given at Q2W or Q4W dosing schedules, and large dosages are required to sustain LDL-C reductions when administered at Q4W. It has been reported that 76.9% of patients were fully adherent to bi-weekly alirocumab or evolocumab in the real-world, defined as taking at least 80% of the prescribed medication, which was superior to stains [26] but still might be improved by less frequent subcutaneous dosing. Until December 2020, the European Commission approved inclisiran (a small interfering RNA targeting hepatic PCSK9 synthesis) with an infrequent dosing for heterozygous familial hypercholesterolemia (starting with an initial dose, then administered again at 3 months and then every 6 months thereafter), based on an LDL-C reduction of −47.9% [27]. We also designed this study aiming to assess recaticimab with infrequent dosing schedules. The LS mean percentage reductions in LDL-C from baseline to end of treatment were −52.02% and −48.38% with recaticimab at 150Q8W and 300Q8W, and −43.93% and −52.77% with recaticimab at 300Q12W and 450Q12W (all *p* values <0.0001 as compared with the placebo group). Although there was a slight rebound before the next administration, substantial reductions in LDL-C level were observed during whole treatment period. The percentage of patients who fully adhered to recaticimab ranged from 95.0 to 100% at different dosing schedules, making high compliance with recaticimab in clinical practice worthy to be awaited.

Except LDL-C, reductions in TC, non-HDL-C, ApoB, and Lp(a) with recaticimab treatment were also rapid and substantial, similar to other monoclonal antibodies against PCSK9. Established evidence supports that increased LDL-C and TC levels are causally related to atherosclerotic cardiovascular disease events and mortality [1, 3, 28]. Non-HDL-C stands for an alternative calculated LDL-C, and ApoB concentration is very highly correlated with LDL-C level. ApoB-containing lipoproteins in the arterial wall could cause lipid deposition and then the initiation, accumulate, growth, and progression of atherosclerotic plaques, increasing the risk of atherosclerotic cardiovascular disease events [29]. If available, ApoB could be considered as an alternative risk marker. In addition, despite weaker than LDL-C, elevated Lp(a) is an independent risk factor for atherosclerotic cardiovascular disease [30–32]. PCKS9 inhibitors resulted in a reduction in Lp(a) level to 25–30%, and reduction in Lp(a) level was found to be associated with a lower risk of major adverse cardiovascular events after adjustment for baseline level [33]. Consequently, recaticimab may offer the potential for enhanced cardiovascular benefits by lowering LDL particles and these atherogenic lipoproteins, which needs to be proven by long-term follow-up.

Sharp percentage reduction in circulating free PCSK9 was detected immediately after administration of recaticimab, but rebound was observed before next administration, which may be caused by background atorvastatin use [22, 23]. However, of note, despite the high variation of PCSK9, no obvious effect on efficacy of recaticimab was observed.

Compared with other monoclonal antibodies against PCSK9, the half-life of recaticimab was relatively longer than that of alirocumab and evolocumab (recaticimab: 18.6–27.4 days; alirocumab: 17–20 days; evolocumab: 11–17 days) [34, 35], which may be caused by different binding epitope of recaticimab that contains YTE mutation, an FcRn affinity-enhancing Fc mutant [36]. The long half-life supported the substantial reductions in LDL-C level with recaticimab therapy, even with an infrequent dosing.

Incidences of TEAEs were similar between recaticimab and placebo. The majority of TEAEs with recaticimab were mild, and almost all moderate and severe treatment-related TEAEs can be relieved. No serious TEAEs, fatal TEAE, or TEAE leading to treatment termination occurred, and no clinically relevant safety issues emerged. The TEAE profile was similar to other PCSK9 inhibitors. This study was limited by a short follow-up duration. Long-term safety data and effects on cardiovascular outcomes with recaticimab therapy are being collected.

## Conclusion

Our findings supported the use of recaticimab, even given once every 12 weeks, as add-on therapy to stable dose of a statin in patients with hypercholesterolemia, providing a new safe and effective treatment option for this population. Large-scale phase 3 studies are being planned for recaticimab monotherapy or combination therapy.

## Supplementary Information

**Additional file 1: Figure S1.** Study design. **Figure S2.** Percentage change in TC during treatment. **Figure S3.** Percentage change in non-HDL-C during treatment. **Figure S4.** Percentage change in ApoB during treatment. **Figure S5.** Percentage change in Lp(a) during treatment. **Figure S6.** Percentage change in HDL-C during treatment. **Figure S7.** Percentage change in TG during treatment. **Figure S8.** Percentage change in ApoA1 during treatment. **Figure S9.** Percentage change in free PCSK9 during treatment. **Figure S10.** Serum concentration-time profile of recaticimab. **Figure S11.** Serum exposure of recaticimab after the first dose (**A**) and last dose (**B**) according to the presence or absence of ADAs. **Table S1.** Baseline characteristics of patients. **Table S2.** Moderate or severe treatment-emergent adverse events in recaticimab groups. **Full inclusion** and **exclusion criteria**.

## Data Availability

The data that support the findings of this study are available on request from the corresponding authors after the completion of the study. The data are not publicly available due to privacy or ethical restrictions.

## Abbreviations

LDL-C: Low-density lipoprotein-cholesterol
PCSK9: Proprotein convertase subtilisin/kexin type 9
TG: Triglyceride
TEAEs: Treatment-emergent adverse events
T_max_: Maximum serum concentration
C_max_: Maximum serum concentration
AUC_0-τ_: Area under the plasma concentration-time profile from time zero to the treatment
AUC_last_: Area under the plasma concentration-time profile from time zero to the last concentration measurement
AUC_0-∞_: Area under the plasma concentration-time profile from time zero to infinity
t_1/2_: Half-life
CL/F: Apparent total clearance
V_z_/F: Apparent volume of distribution
C_trough_: Trough concentration
SD: Standard deviation
LS: Least-squares
CIs: Confidence intervals
