# Supplementary material for: PCSK9 inhibitor recaticimab for hypercholesterolemia on stable statin dose: a randomized, double-blind, placebo-controlled phase 1b/2 study
Source: BMC Med. 2022 Jan 18;20:13. doi: 10.1186/s12916-021-02208-w (PMC8763618; doi:10.1186/s12916-021-02208-w)
Supplement: Supplementary file 1 — Additional file 1: Figure S1. Study design. Figure S2. Percentage change in TC during treatment. Figure S3. Percentage change in non-HDL-C during treatment. Figure S4. Percentage change in ApoB during treatment. Figure S5. Percentage change in Lp(a) during treatment. Figure S6. Percentage change in HDL-C during treatment. Figure S7. Percentage change in TG during treatment. Figure S8. Percentage change in ApoA1 during treatment. Figure S9. Percentage change in free PCSK9 during treatment. Figure S10. Serum concentration-time profile of recaticimab. Figure S11. Serum exposure of recaticimab after the first dose (A) and last dose (B) according to the presence or absence of ADAs. Table S1. Baseline characteristics of patients. Table S2. Moderate or severe treatment-emergent adverse events in recaticimab groups. Full inclusion and exclusion criteria. [file 12916_2021_2208_MOESM1_ESM.docx]

**Supplementary**

**Table of Contents**

[Figure S1. Study design 2](#_Toc85814098)

[Figure S2. Percentage change in TC during treatment 3](#_Toc85814099)

[Figure S3. Percentage change in non-HDL-C during treatment 3](#_Toc85814100)

[Figure S4. Percentage change in ApoB during treatment 4](#_Toc85814101)

[Figure S5. Percentage change in Lp(a) during treatment 4](#_Toc85814102)

[Figure S6. Percentage change in HDL-C during treatment 5](#_Toc85814103)

[Figure S7. Percentage change in TG during treatment 5](#_Toc85814104)

[Figure S8. Percentage change in ApoA1 during treatment 6](#_Toc85814105)

[Figure S9. Percentage change in free PCSK9 during treatment 6](#_Toc85814106)

[Figure S10. Serum concentration-time profile of recaticimab 7](#_Toc85814107)

[Figure S11. Serum exposure of recaticimab after the first dose (A) and last dose (B) according to the presence or absence of ADAs 8](#_Toc85814108)

[Table S1. Baseline characteristics of patients 9](#_Toc85814109)

[Table S2. Moderate or severe treatment-emergent adverse events in recaticimab groups 10](#_Toc85814110)

[Full inclusion and exclusion criteria 11](#_Toc85814111)

# Figure S1. Study design


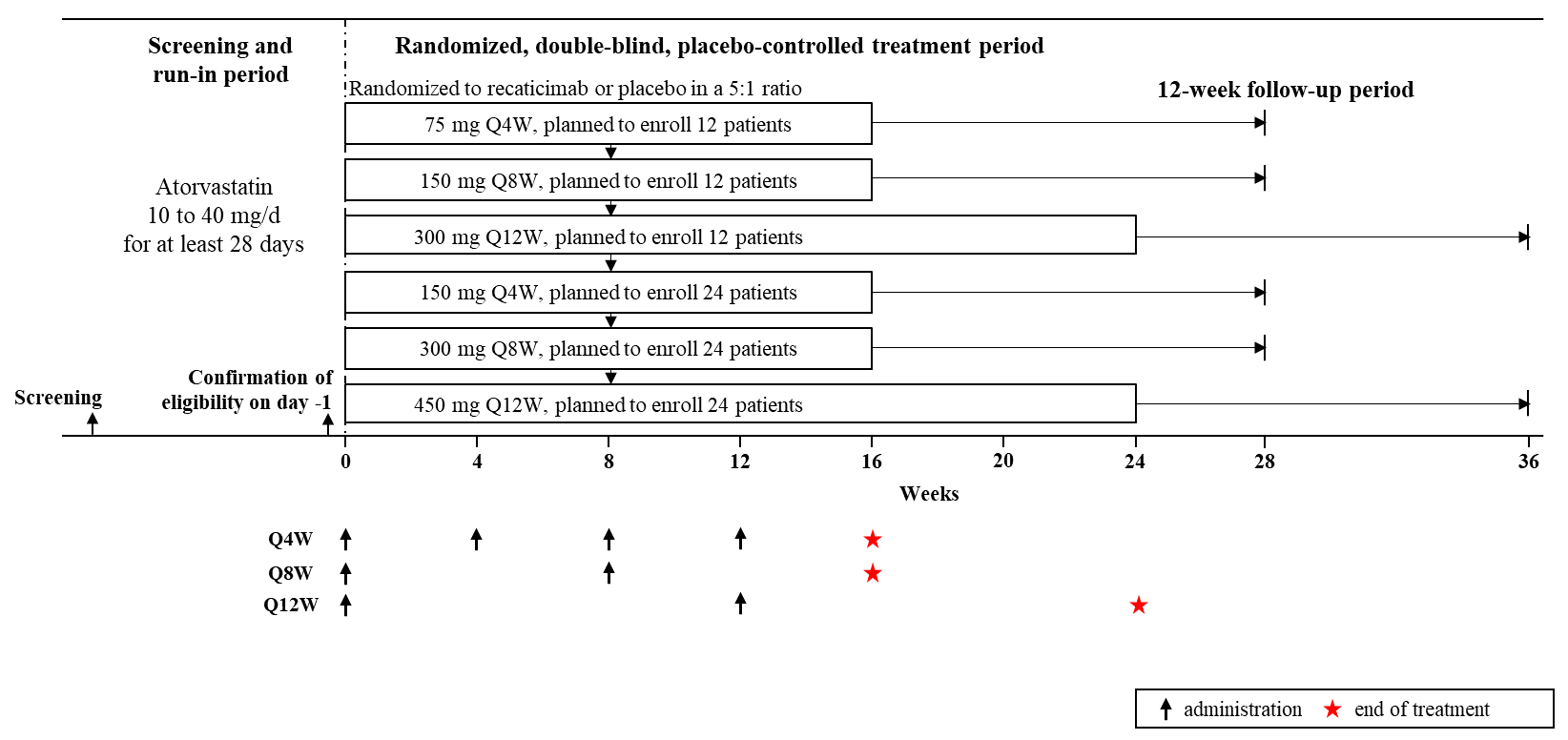


This study included a screening and statin run-in period, a treatment period, and a 12-week follow-up period. All patients were required to receive a stable regimen of atorvastatin (10 to 40 mg/d) for at least 28 days before randomization and continue the regimen throughout the study. Patients who had been on the prescribed atorvastatin for at least 28 days before screening directly entered the treatment period. Patients who had not previously received the prescribed atorvastatin or who had not received the prescribed atorvastatin over 28 days entered the statin run-in period.

# Figure S2. Percentage change in TC during treatment


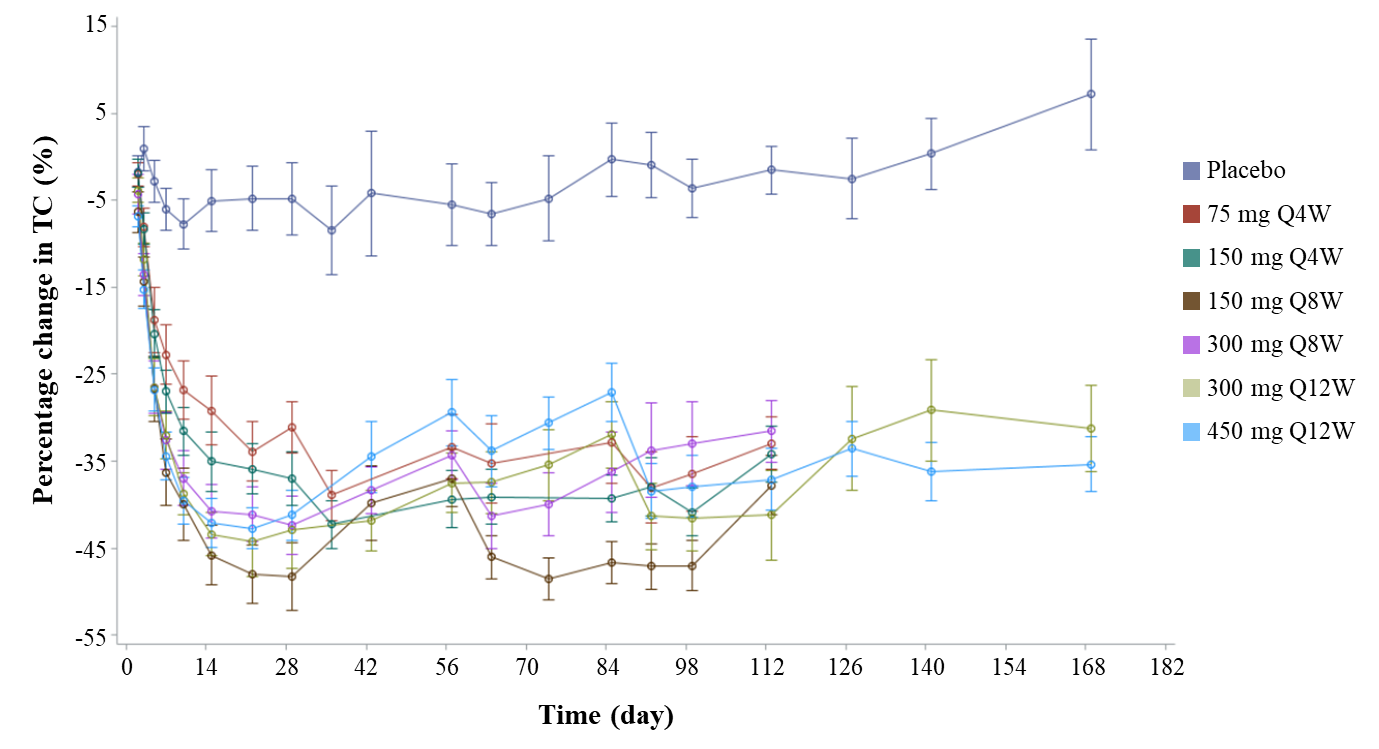


Data are mean (standard error). TC, total cholesterol.

# Figure S3. Percentage change in non-HDL-C during treatment


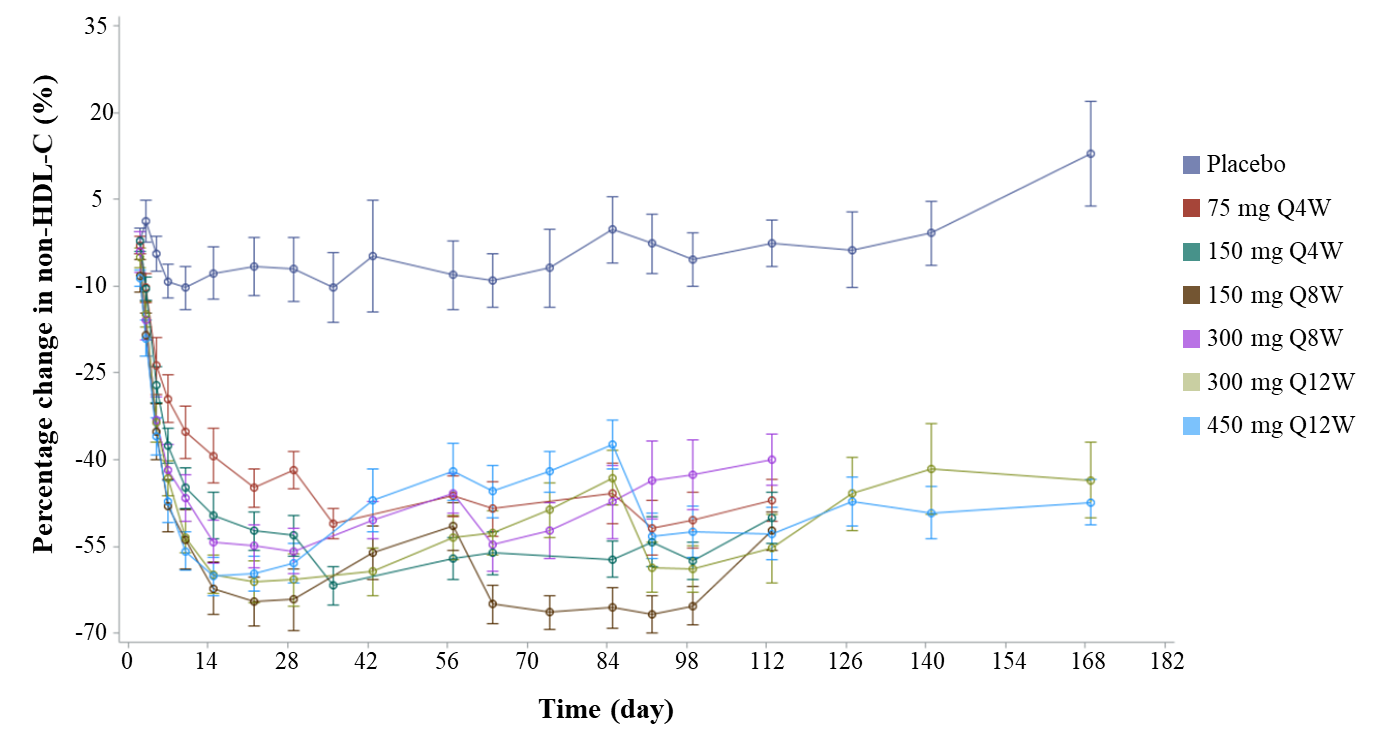


Data are mean (standard error). non-HDL-C, non-high-density lipoproteincholesterol.

# Figure S4. Percentage change in ApoB during treatment


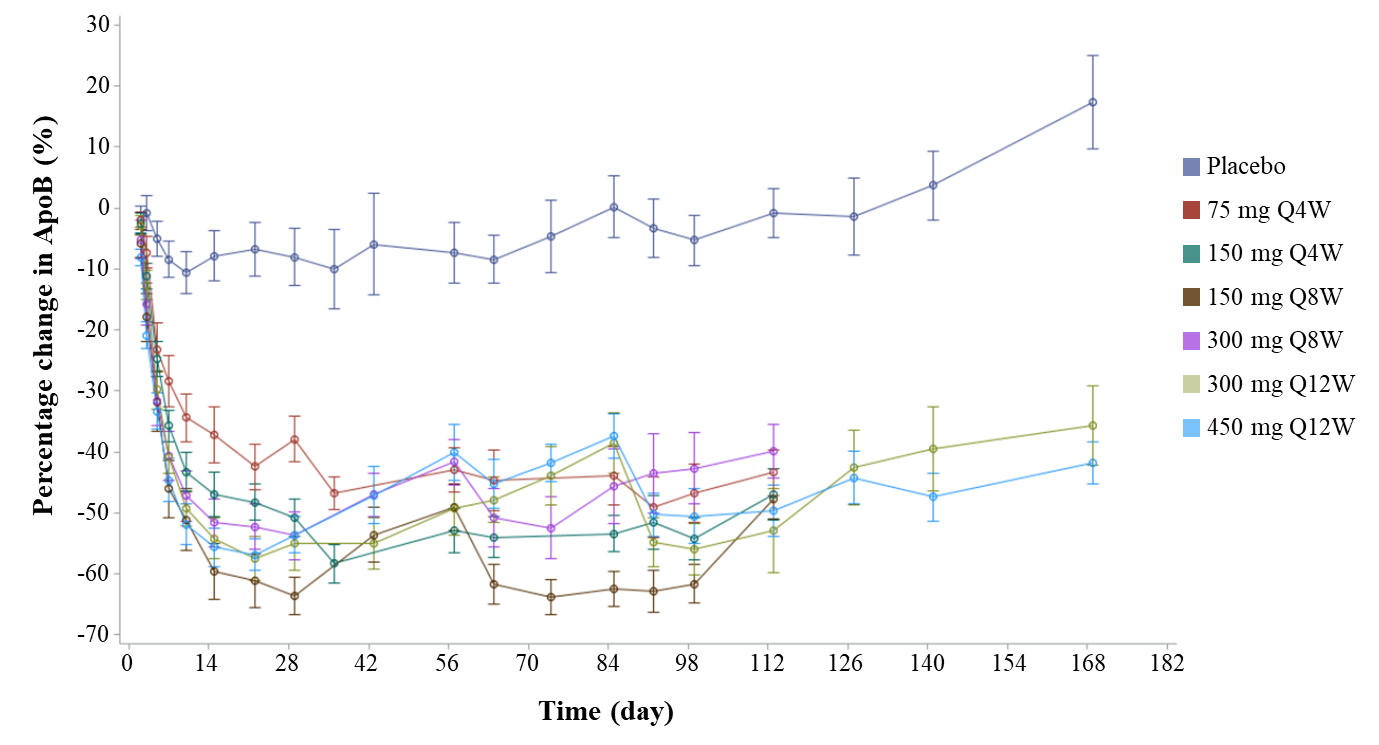


Data are mean (standard error). ApoB, apolipoprotein B.

# Figure S5. Percentage change in Lp(a) during treatment


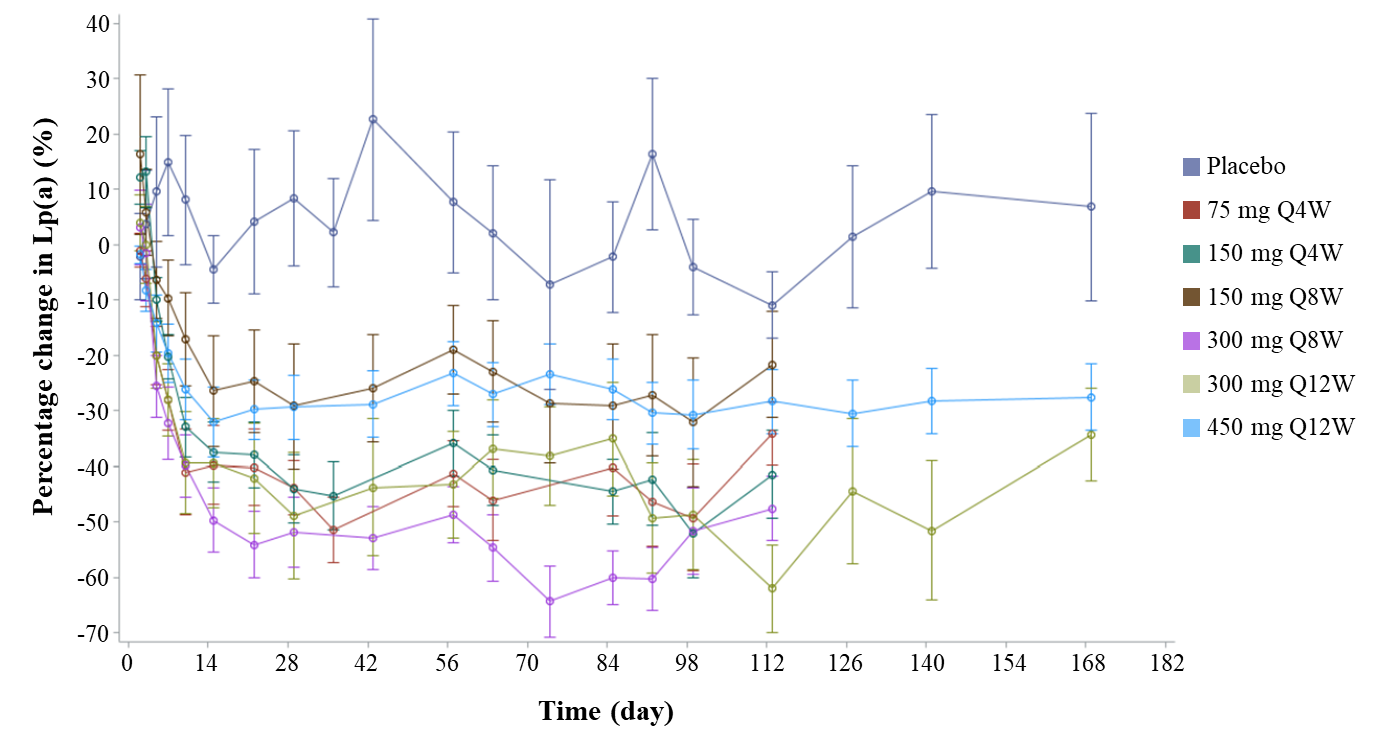


Data are mean (standard error). Lp(a), apolipoprotein (a).

# Figure S6. Percentage change in HDL-C during treatment


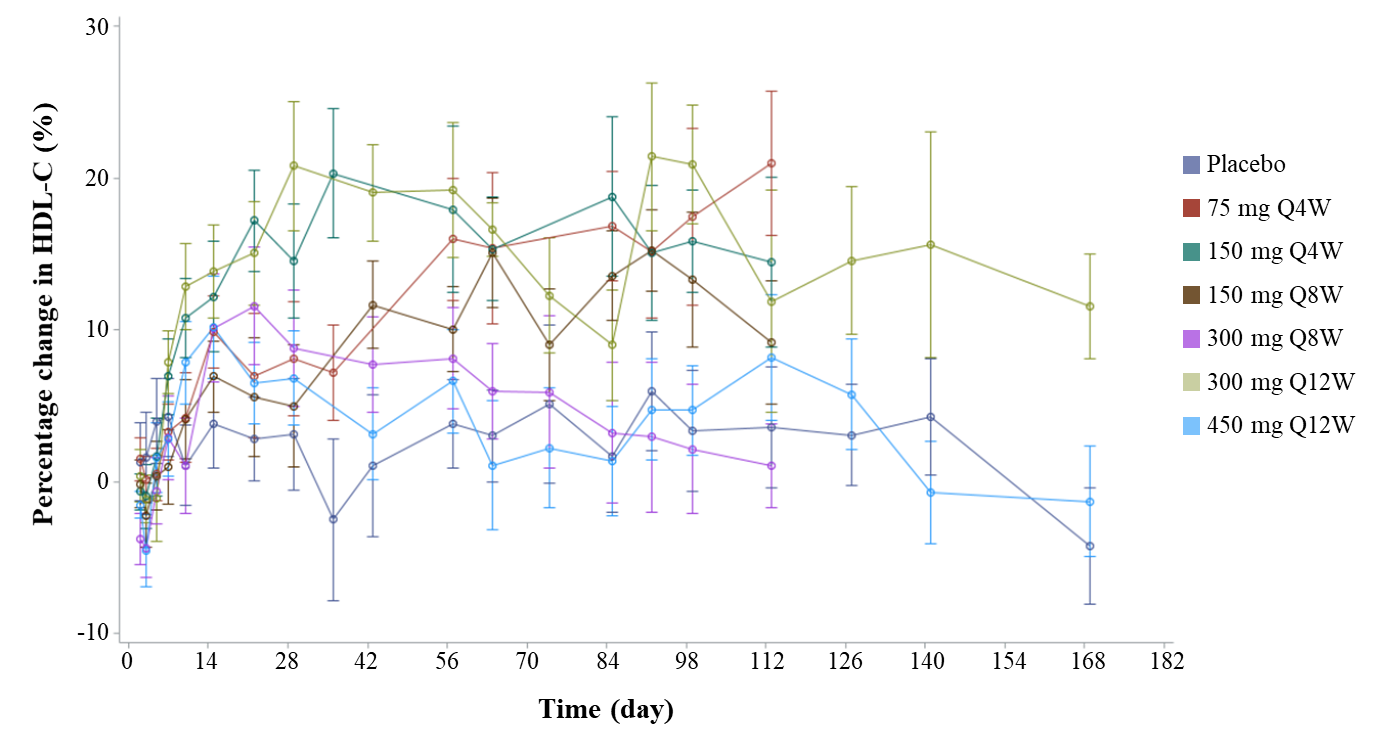


Data are mean (standard error). HDL-C, high-density lipoproteincholesterol.

# Figure S7. Percentage change in TG during treatment


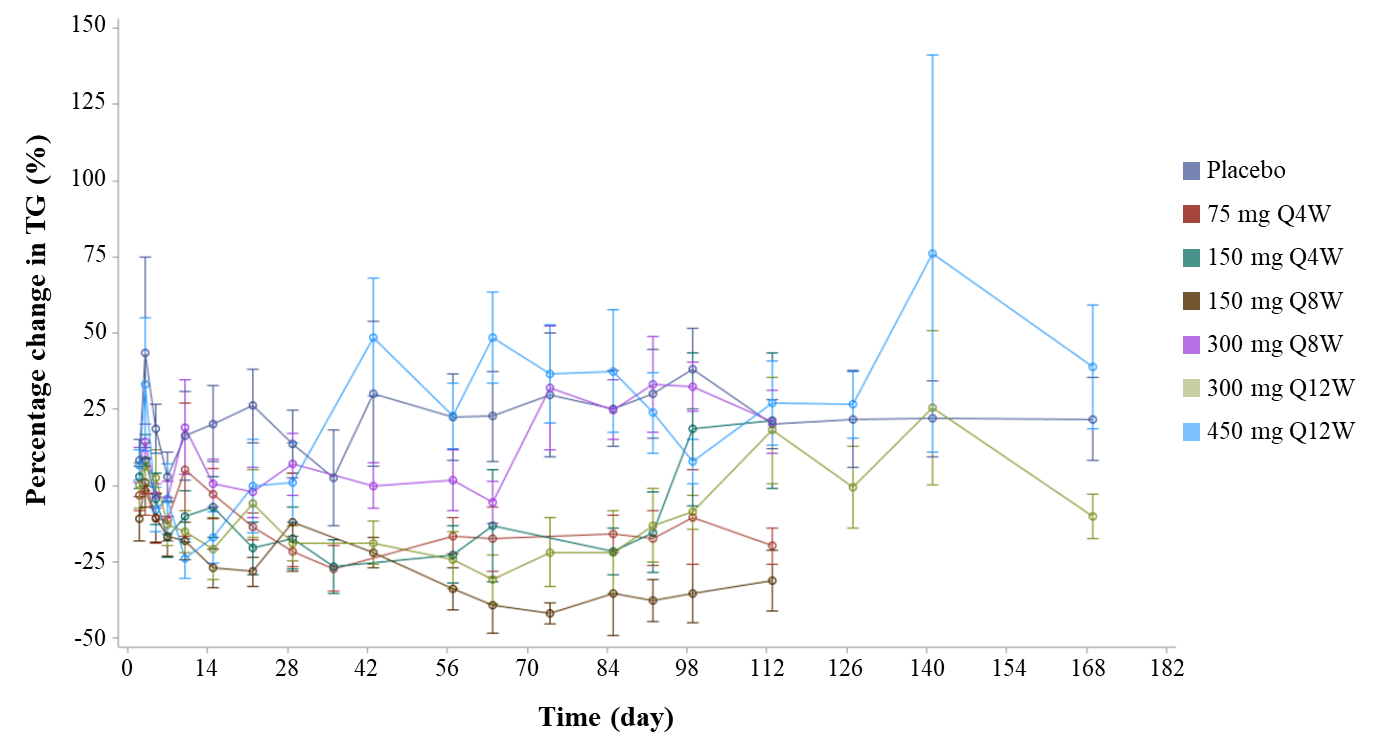


Data are mean (standard error). TG, triglyceride.

# Figure S8. Percentage change in ApoA1 during treatment


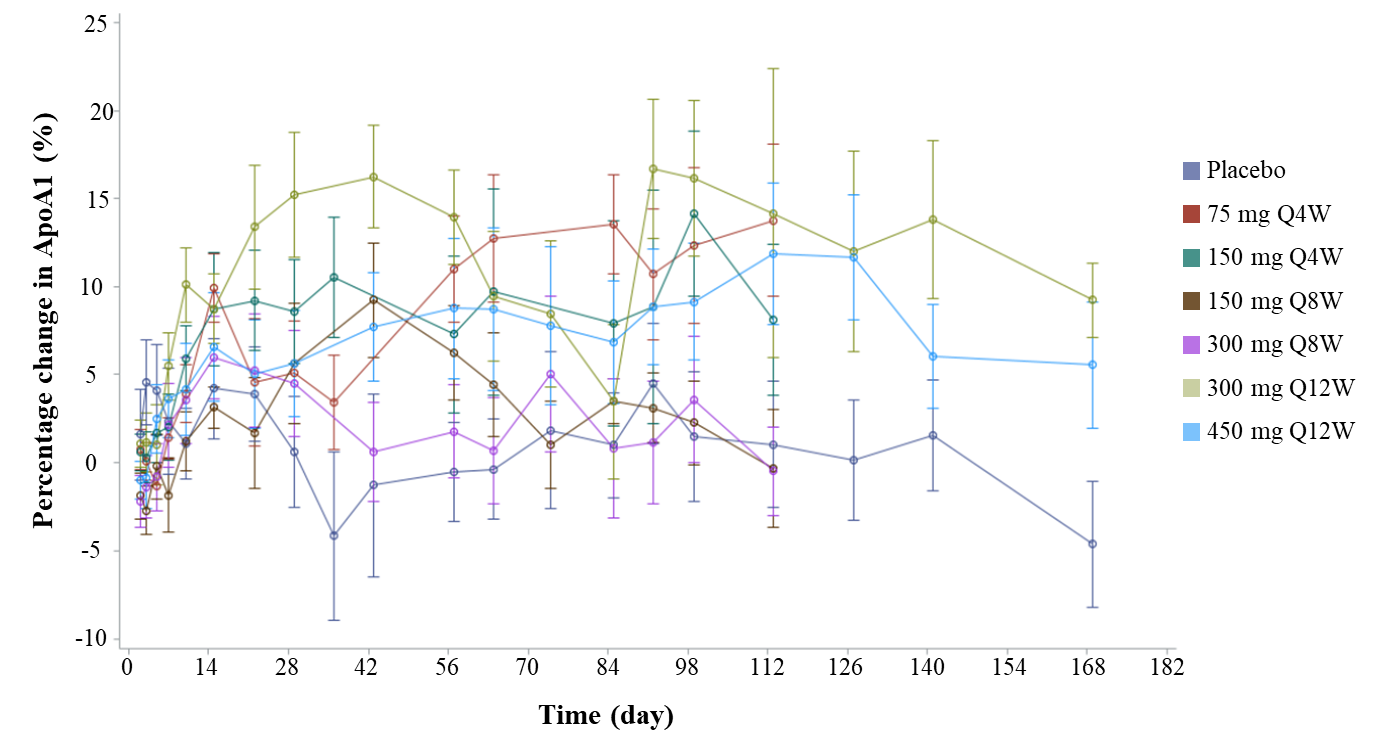


Data are mean (standard error). ApoA1, apolipoprotein A1.

# Figure S9. Percentage change in free PCSK9 during treatment

**
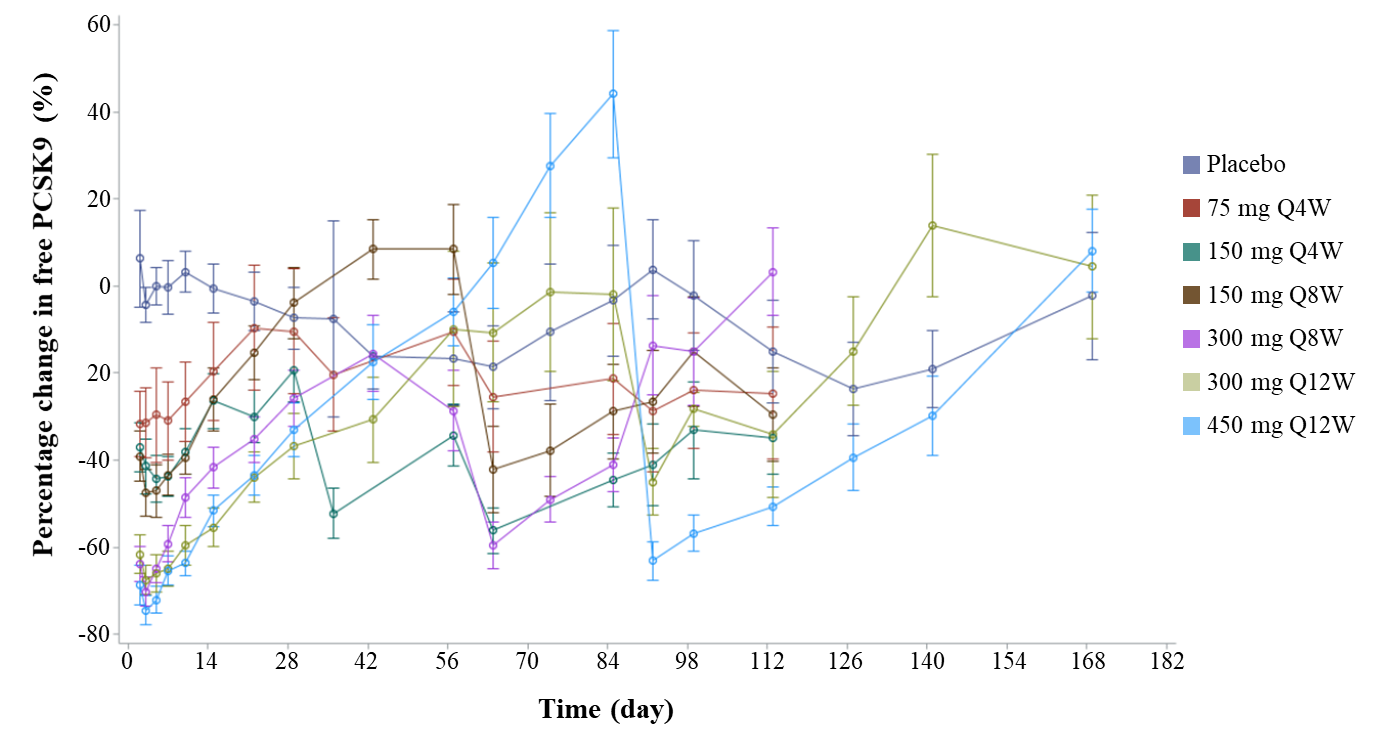
**

Data are mean (standard error). PCSK9, proprotein convertase subtilisin/kexin type 9 serine protease.

# Figure S10. Serum concentration-time profile of recaticimab

**
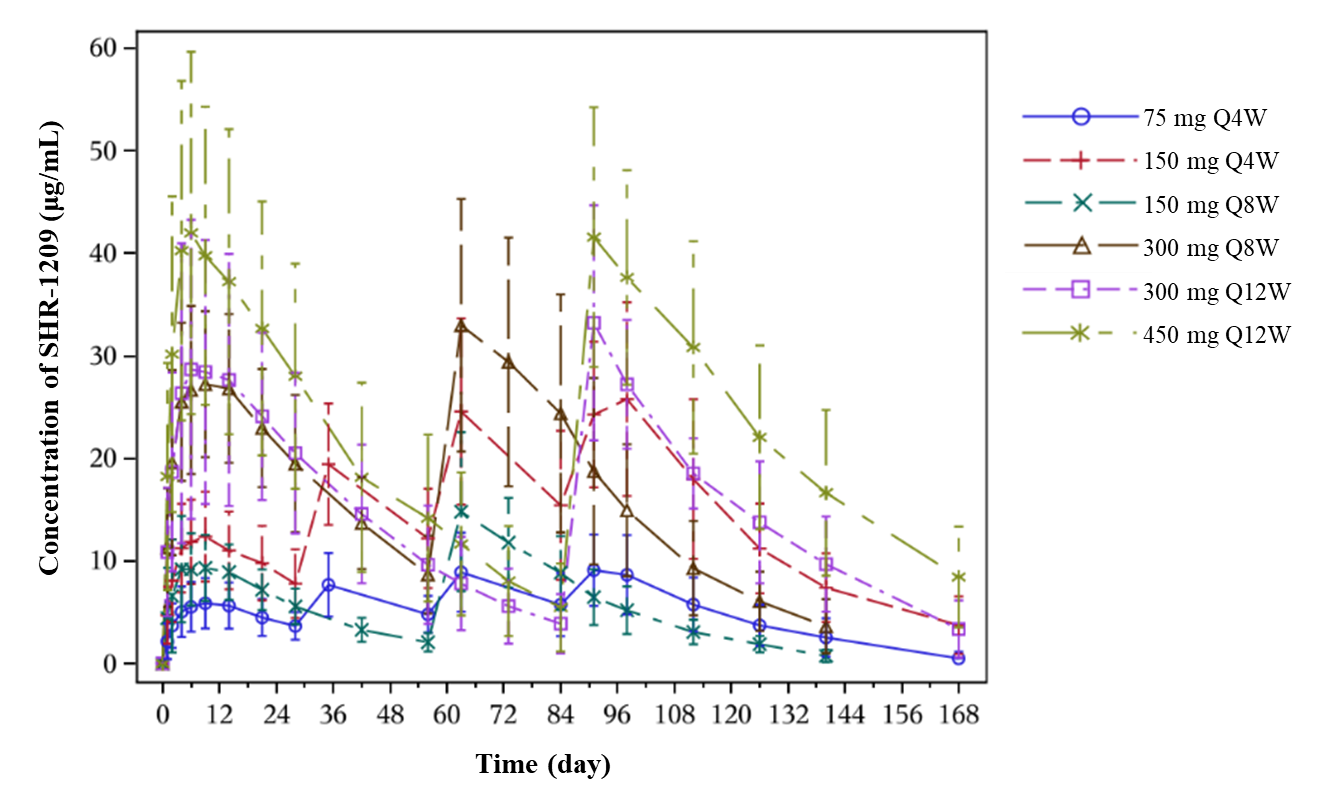
**

Data are mean (standard deviation). A total of 91 patients who received at least one dose of the study treatment and had evaluable post-treatment pharmacokinetic data were included.

# Figure S11. Serum exposure of recaticimab after the first dose (A) and last dose (B) according to the presence or absence of ADAs

**
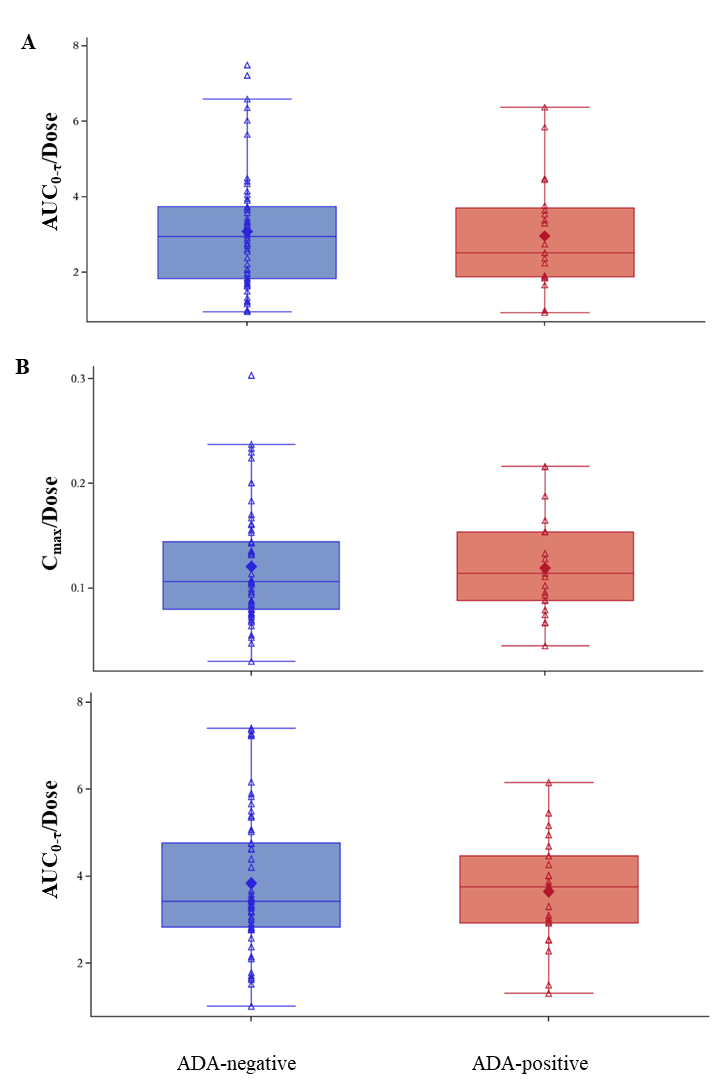
**

Data are median (interquartile range). ADA, antidrug antibody. C_max_, maximum serum concentration; AUC_0-τ_, area under the plasma concentration-time profile from time zero to the treatment.

# Table S1. Baseline characteristics of patients

|  | **Placebo (N=19)** | **Recaticimab** | | | | | | | **All patients (N=110)** |
| --- | --- | --- | --- | --- | --- | --- | --- | --- | --- |
|  |  | **75 mg**  **Q4W**  **(N=10)** | **150 mg**  **Q4W**  **(N=20)** | **150 mg**  **Q8W**  **(N=10)** | **300 mg**  **Q8W**  **(N=20)** | **300 mg**  **Q12W**  **(N=10)** | **450 mg**  **Q12W**  **(N=21)** | **All patients with recaticimab (N=91)** |  |
| **Age (years)** | 53.6 (10.3) | 45.2 (10.6) | 46.8 (7.5) | 55.8 (4.2) | 50.0 (9.3) | 49.5 (8.3) | 51.0 (10.9) | 49.5 (9.2) | 50.3 (9.5) |
| **Gender, n (%)** | | | | | | | | | |
| Male | 7 (36.8%) | 5 (50.0%) | 12 (60.0%) | 5 (50.0%) | 10 (50.0%) | 8 (80.0%) | 11 (52.4%) | 51 (56.0%) | 58 (52.7%) |
| Female | 12 (63.2%) | 5(50.0%) | 8 (40.0%) | 5 (50.0%) | 10 (50.0%) | 2 (20.0%) | 10 (47.6%) | 40 (44.0%) | 52 (47.3%) |
| **BMI (kg/m^2^)** | 25.01 (3.51) | 25.25 (3.63) | 25.13 (2.94) | 26.50 (2.54) | 26.24 (3.85) | 25.04 (2.55) | 26.31 (4.33) | 25.80  (3.48) | 25.66  (3.48) |
| **Cardiovascular history and risk factors, n (%)** | | | | | | | | | |
| **Type 2 diabetes** | 3 (15.8%) | 0 (0%) | 2 (10.0%) | 2 (20.0%) | 3 (15.0%) | 1 (10.0%) | 4 (19.0%) | 12 (13.2%) | 15 (13.6%) |
| **Hypertension** | 8 (42.1%) | 3 (30.0%) | 10 (50.0%) | 3 (30.0%) | 7 (35.0%) | 4 (40.0%) | 7 (33.3%) | 34 (37.4%) | 42 (38.2%) |
| **Smoking history** | 6 (31.6%) | 2 (20.0%) | 5 (25.0%) | 0 (0%) | 5 (25.0%) | 4 (40.0%) | 8 (38.1%) | 24 (26.4%) | 30 (27.3%) |
| **ASCVD** | 1 (5.3%) | 0 (0%) | 1 (5.0%) | 2 (20.0%) | 1 (5.0%) | 1 (10.0%) | 2 (9.5%) | 6 (6.6%) | 7 (6.4%) |

Values are mean (standard deviation) unless otherwise noted.

BMI, body mass index; ASCVD, atherosclerotic cardiovascular disease.

# Table S2. Moderate or severe treatment-emergent adverse events in recaticimab groups

|  | **All-causality**  **(N=91)** | **Treatment-related**  **(N=91)** |
| --- | --- | --- |
| **Moderate treatment-emergent adverse events** | **5 (5.5%)** | **3 (3.3%)** |
| Alanine aminotransferase increased | 1 (1.1%) | 1 (1.1%) |
| Gamma-glutamyltransferase increased | 1 (1.1%) | 1 (1.1%) |
| Aspartate aminotransferase increased | 1 (1.1%) | 1 (1.1%) |
| Herpes simplex | 1 (1.1%) | 1 (1.1%) |
| Ventricular extrasystoles | 1 (1.1%) | 1 (1.1%) |
| Blood glucose increased | 1 (1.1%) | 0 |
| Upper respiratory tract infection | 1 (1.1%) | 0 |
| Hyperglycaemia | 1 (1.1%) | 0 |
| Abdominal pain upper | 1 (1.1%) | 0 |
| Toothache | 1 (1.1%) | 0 |
| Anaemia | 1 (1.1%) | 0 |
| **Severe treatment-emergent adverse events** | **1 (1.1%)** | **1 (1.1%)** |
| Gamma-glutamyltransferase increased | 1 (1.1%) | 1 (1.1%) |

Data are shown in n (%).

# Full inclusion and exclusion criteria

**1. Inclusion criteria**

Patients should meet all the following criteria.

1. The age of patients when signing the informed consent form must be between 18 to 65 years; both male and female are eligible;
2. If patients are receiving or eligible for any statin treatment, they are required to receive a stable regimen of atorvastatin (10 to 40 mg/d) for at least 28 days before randomization and continue the regimen throughout this study;
3. Patients should have a low-density lipoprotein-cholesterol (LDL-C) level of 2.6 mmol/L or higher before randomization (either for those receiving statin therapy and/or other lipid-lowering treatments and had an LDL-C level of 2.6 mmol/L or higher at screening, or for those who had not previously received any lipid-lowering treatment and had an LDL-C level of 3.4 mmol/L or higher at screening);
4. Having a fasting triglyceride (TG) level of 4.5 mmol/L or lower;
5. Having a body mass index of 18 to 35 kg/m^2^;
6. Patients should understand the study procedures and methods, voluntarily participate in this study, and sign the written informed consent.

**2. Exclusion criteria**

Patients should not meet any of the following criteria.

1. Having the following diseases or treatment history:
   1. be allergic to the study drug or any component in the study drug, or had a severe allergic reaction to other antibody drugs;
   2. diagnosis of homozygous familial hypercholesterolemia according to the European Society of Cardiology Guidelines;
   3. prior therapy with SHR-1209 or other inhibitors targeting PCSK9;
   4. a history of New York heart association class II-IV heart failure;
   5. a history of acute coronary syndrome (such as myocardial infarction and unstable angina), percutaneous coronary intervention, or coronary artery bypass graft within 12 months before screening; or a history of cerebrovascular disease (such as stroke and transient ischemic attack) within 12 months before screening;
   6. uncontrolled severe arrhythmias, defined as recurrent and highly symptomatic ventricular tachycardia and atrial fibrillation with rapid ventricular response or supraventricular tachycardia that are not controlled by drugs or other therapies within 12 months before enrollment;
   7. uncontrolled hypertension, defined as blood systolic blood pressure ≥160 and/or diastolic blood pressure ≥100 mmHg;
   8. uncontrollable diseases affecting blood lipid levels, such as nephrotic syndrome, severe liver disease, Cushing’s syndrome, and abnormal thyroid function (Patients with hypothyroidism before screening can be enrolled, if they have been treated with stable thyroid hormone replacement therapy for at least 28 days, have normal level of thyroid-stimulating hormone, and agree to continue thyroid hormone replacement therapy with the stable dose during this study);
   9. type 1 diabetes; poorly controlled type 2 diabetes (hemoglobin A1c, higher than 8.5%), or type 2 diabetes being treated with or required treatment with insulin or glucagon-like peptide-1 receptor agonist;
   10. unstable or severe liver, kidney, digestive tract, neuropsychiatric, and hematological diseases, or abnormal laboratory tests, that may lead to unacceptable risk judged by the investigator;
   11. a history of malignant tumor or lymphoproliferative diseases;
   12. participated in other drug or medical device clinical trial within one month before screening, or have stopped treatment but still within 5 half-lives of the drug at screening (for drugs with 5 half-lives over 1 month);
   13. severe trauma or surgery within 6 months before screening, or plan to undergo surgery during this study;
   14. a history of blood donation within 3 months before screening, blood loss of 400 mL or more, or received blood transfusion within 3 months before screening;
   15. received a live-attenuated vaccine within 3 months before screening or plan to receive during this study;
   16. a history of drug abuse, substance abuse, or alcoholism.
2. Having the following treatment history with 4 weeks before screening:
   1. systemic corticosteroids, including but not limited to intra-articular corticosteroids regularly used in veins and muscles;
   2. drugs, nutraceuticals, and Chinese patent medicines with an indication of weight loss (such as orlistat);
   3. diuretics, including but not limited to loop diuretics, thiazide diuretics, potassium sparing diuretics, carbonic anhydrase inhibitors;

Patients who have stopped the above drugs before screening can be included after a sufficient washout period.

- 1. receiving and unwilling to stop lipid-lowering drugs or nutraceuticals other than atorvastatin, including but not limited to ezetimibe, fibrates, niacin, omega-3 fatty acids or bile acid sequestrants.

1. Any laboratory test meets the following criteria during screening or at baseline:
   1. estimated glomerular filtration rate <45 ml/min/1.73 m^2^ at screening based on Renal Disease Study Formula;
   2. alanine aminotransferase, aspartate aminotransferase, or γ-glutamyltransferase > 2.5 times the upper limit of normal (×ULN) at screening;
   3. creatine kinase > 3×ULN at screening;
   4. positive for human immunodeficiency virus, syphilis serology, or hepatitis C virus; positive for hepatitis B surface antigen with hepatitis B virus DNA ≥1000 copies/ml;
   5. women with a positive blood pregnancy test at screening or baseline.
2. Others:
   1. patients with major lifestyle changes within 4 weeks before screening or or who cannot maintain the lifestyle during this study;
   2. women of childbearing age who do not use contraception within 4 weeks before the administration of study treatment; men or women who do not agree to use effective contraception during this study or within 24 weeks after the last dose of study treatment;
   3. pregnant and breastfeeding women; women who plan to become pregnant during this study or within 24 weeks after the last dose of study treatment;
   4. any other conditions that are not suitable to participate in this study at the discretion of the investigator.
